# Effect of the Virtual Reality-Infused Movement and Activity Program (V-MAP) on Physical Activity and Cognition in Head Start Preschoolers

**DOI:** 10.3390/children12091228

**Published:** 2025-09-14

**Authors:** Xiangli Gu, Samantha Moss, Xiaoxia Zhang, Tao Zhang, Tracy L. Greer

**Affiliations:** 1 Department of Kinesiology, The University of Texas at Arlington, Arlington, TX 76019, USA; 2 Department of Kinesiology, Health Promotion, and Recreation, University of North Texas, Denton, TX 76203, USA; samantha.moss@unt.edu (S.M.); tao.zhang@unt.edu (T.Z.); 3 School of Sport Sciences, West Virginia University, Morgantown, WV 26505, USA; xiaoxia.zhang@mail.wvu.edu; 4 Department of Psychology, The University of Texas at Arlington, Arlington, TX 76019, USA; tracy.greer@uta.edu

**Keywords:** fundamental movement skill, cognitive function, virtual-reality intervention, physical activity

## Abstract

**Highlights:**

**What are the main findings?**

• V-MAP significantly increased vigorous physical activity by 10 min per day during the school day.
• V-MAP did not result in significant immediate cognitive gains, but delayed effects were seen at one-month follow-up.

**What is the implication of the main finding?**

• Implementing V-MAP in recess may help Head Start preschoolers achieve or accumulate the recommended daily 60-minute MVPA guideline.
• VR-based physical activity for as little as 30 min per day may benefit cognitive capability.

**Abstract:**

**Background/Objectives:** This study examined the efficacy of a physical activity (PA) intervention augmented by a non-immersive Virtual Reality (VR) gaming system (i.e., Virtual Reality-infused Movement and Activity Program; V-MAP) on physical activity (i.e., sedentary behavior, moderate-to-vigorous PA [MVPA], vigorous PA [VPA]) and cognitive skills (i.e., response error, movement latency and reaction time) in Head Start preschoolers. Methods: Using a repeated-measure with 1-month follow-up design, a sample of 13 Head Start preschoolers (Mage = 67.08 ± 4.32 months; 36.2% boys) engaged in a 6-week V-MAP intervention (30-min session; 8 sessions) that focused on non-immersive VR based movement integration. The Cambridge Neuropsychological Test Automated Battery (CANTAB) was used to measure cognition; school-based PA and sedentary behavior were assessed by ActiGraph accelerometer. Pedometers were used to monitor real time engagement and implementation over eight intervention sessions. **Results**: On average, children obtained 1105 steps during the 30-min intervention (36.85 steps/min). There was a significant increase in VPA after the V-MAP intervention, whereas no significant changes in MVPA or sedentary behavior were observed (ps > 0.05). Although we did not observe significant improvement in studied cognitive function variables (ps > 0.05) after the V-MAP intervention, some delayed effects were observed in the follow-up test (Cohen’s d ranges from −0.41 to −0.73). **Conclusions**: This efficacy trial provides preliminary support that implementing V-MAP in recess may help Head Start preschoolers achieve or accumulate the recommended daily 60-min MVPA guideline during preschool years. The findings also provide insights that VR-based PA for as little as 30 min per day may benefit cognitive capability.

## 1. Introduction

One third of Head Start preschoolers are either overweight or obese and demonstrate increased risk of developmental delays, especially in disadvantaged Hispanic and African American communities [1,2]. Only 50–54% of children between the ages of 2–6 meet the 60-min per day moderate-to-vigorous physical activity (MVPA) recommendation [3,4,5] and an estimated 15% of children in the United States have at least one development delay [6]. The challenges associated with the COVID-19 pandemic will have a lasting impact for minority and economically disadvantaged Head Start preschoolers because fundamental health disparities persist, further inhibiting already limited access to community resources and active learning environments. Head Start preschoolers show an increased risk of motor, cognitive and social-emotional development delays and the overweight/obese ratio is much higher than the national average [1,7], thus obesity prevention in this vulnerable population has great potential for long-term impact. Elevated risk of obesity and the exacerbated social and health inequities have positioned Head Start preschoolers in the vulnerable developmental trajectory, thus behavioral interventions focusing on physical activity promotion during early childhood are greatly needed.

CDC data demonstrated that physical activity for as little as 5–10 min per day may benefit cognitive function such as increased concentration and attention [8,9,10]. Previous studies [11,12] have reported significant improvements in Head Start preschoolers’ physical activity levels and development (i.e., gross motor skills) through physical activity interventions focused on enhancing motor skills. One study [13] in particular demonstrated that pairing physical activity and fundamental movement skill practice in their 7-week recess intervention yielded greater daily MVPA levels for all Head Start preschoolers compared to the groups engaging in only physical activity or only fundamental movement skills. Recess offers tremendous opportunities for children to accumulate physical activity and to practice various fundamental movement skills [14,15,16].

Research suggests that interventions during recess breaks are more cost-effective compared with other school-based intervention programs for physical activity promotion [17]. Specifically, research has shown that young children (e.g., kindergarteners) spend more time doing on-task behaviors in the classroom when engaged in active recess or active classroom breaks. Children can accumulate up to 40% of their total daily physical activity during structured recess alone where their energy expenditure can reach 100 kcal/30 min, which is more intensive than in unstructured recess [18,19]. Research also supports the notion that time spent in recess improves children’s academic behaviors, such as increasing on-task behaviors, reducing disruptive behaviors, and improving classroom behaviors [8,20,21,22].

It was noticed that recess-based interventions may work better when relevant modern technology is infused (e.g., wearable devices and video gaming). Technology-enabled movement integration (MI) is a promising strategy to improve movement and classroom behaviors, but more empirical evidence is needed [23,24]. Researchers have used technology, such as video games and wearable devices, to increase physical activity and learning outcomes [24,25,26]. A recent study reported that preschoolers who were randomly assigned to an exergaming group demonstrated higher school-day physical activity (pedometer counts) and higher motor competence compared with a free-play group [27]. These results support the use of this modality in childcare settings. For preschoolers with limited motor skill competence, moving/playing with technology can be an attractive approach to build confidence necessary for physical activity engagement. The use of learning technology in young children also improves sensorial perceptual motor-cognitive skills, which may enhance cognitive learning capacity in early school years [28]. More recent intervention studies also suggest that exergaming and technology-based activity programs can increase motivation and physical activity in early childhood populations [24,27].

While some data exist, empirical evidence is limited regarding how technology-infused short bouts of activity in recess and classroom breaks may be associated with improvements in physical activity and cognitive development. Virtual Reality (VR) games can physically and cognitively engage children in fun, short, and full brain-body games that promote cognitive decision making, and movement and reaction skills [29,30,31]. The current study aimed to examine the efficacy of a 6-week physical activity intervention augmented by a non-immersive VR gaming system (i.e., Virtual Reality-infused Movement and Activity Program; V-MAP) on physical activity (i.e., sedentary behavior, MVPA, vigorous physical activity [VPA]) and cognitive function (i.e., movement latency, response errors, and reaction time) in Head Start preschoolers. The outcome variables were measured during three different time points (pre-assessment, post-assessment, and 1-month follow-up-assessment).

## 2. Materials and Methods

### 2.1. Participants

Thirteen participants were recruited from a local Head Start child development center in North Texas. Head Start is a federally funded program in the United States that provides early childhood education, health, nutrition, and parent involvement services to children from low-income families [7]. Eligibility typically includes family income below the federal poverty guideline or other risk factors (e.g., foster care, homelessness). Therefore, children enrolled in Head Start often face socioeconomic disadvantages that may limit access to physical activity opportunities and increase developmental risks. One child moved away prior to the intervention, resulting in a sample of 12 preschoolers (M_age_ = 67.08 ± 4.32 months; 8 girls) who began participation in the intervention. The majority of participants were African American (n =7), followed by 2 White, 2 Asian, and 1 Hispanic. The study was approved by University Institutional Review Board (protocol 2019-0243), and we received the center director’s approval and informed parental consent form for each child prior to conducting baseline assessments and the intervention.

### 2.2. Study Design

The intervention was implemented during school recess time for 6 weeks, totaling 8 intervention sessions (1–2 sessions per week, 30 min/session). Using a repeated-measure with a 1-month follow-up design, preschoolers’ physical activity and cognitive function were assessed before the 6-week intervention and the post-assessments were conducted immediately after the completion of the intervention and follow-up assessments were 1-month after the intervention. To track the children’s engagement and intervention fidelity, each child was asked to wear a numbered pedometer to monitor their step counts generated from each intervention session. The average steps/week were reported in this study.

### 2.3. Virtual Reality-Infused Movement and Activity Program (V-MAP)

The V-MAP intervention was centered on skill-based games augmented by the SMARTfit^TM^ Play Pods (SMARTfit^TM^ Inc., Ventura, CA, USA). The SMARTfit^TM^ system provides a modern, portable, and innovative technology for children to learn while they move and receive instant visual, proprioceptive, tactile, and auditory sensory feedback, including five portable alphanumeric targets (touchable screen) and one CPU (scoring and instruction). Alphanumeric targets displaying colors, numbers, letters, or symbols drive cognitive games such as: Track the Targets (dice, colors, or shapes), Knock the Lights Out, Track the Letters and Numbers, and Seek the Smiley Face, Color, Letter or Number (see Table 1). The VR tasks afford children with active engagement in movement-based games, through which they increase physical activity and promote motor-cognitive skills.

During recess, the children engaged in SMARTfit^TM^-based games/activities for 30 min for 1–2 times/week over 6 weeks (total 8 sessions). Each session implemented 2–3 SMARTfit^TM^ games/activities which was arranged in a logical progression targeting different games by integrating fundamental movement skills: locomotor movements (sliding, skipping, hopping, and running) and object-control movements (throwing, passing, dribbling, striking). Other equipment was integrated to execute object-control movements such as foam noodles and various sized balls. All children participated in the games as one large group or in small groups (depending on the game) with minimal interruption, transition, or waiting time. That is, the different games continue automatically after programming each lesson plan within the software, so the next game will appear on the play pods after striking a “GO” signal. Each game time can be adjusted from 10 s to 5 min. The layout of the strike pods was arranged in a semicircle, circle, or horizontal or staggered line. There were three formation strategies to play the games on the SMARTfit™ system: (1) Partners: Two players are partners and play the game together until time completion (i.e., 10 s/pair) and the next pair of players step up to start a new game with a new time; (2) Team: First set of players plays a turn, retrieves ball/noodle, returns to the start line and hands/passes the ball/noodle to the next player in their line; and (3) Around-the-World: First player takes a turn and peels off to the end of the line. The next player in line plays off the first player’s throw/hand-off and peels off to the end of the line.

The sequence and progress of each V-MAP session took into consideration of age-appropriate levels of difficulty (Table 1 shows the eight V-MAP session plans). The numbered pedometers were assigned to each child during the physical activity intervention and used to track steps and offer immediate visual feedback, such as cumulative step counts, to increase behavioral awareness. The total steps per session were documented to monitor engagement during recess and progress of the V-MAP implementation throughout the intervention period.

### 2.4. Assessment of Physical Activity and Sedentary Behavior

Time spent in different intensities of physical activity and sedentary behavior for 5 consecutive school days were measured using the Actigraph accelerometers (GT3X, ActiGraph Corp, LLC, Pensacola, FL, USA). Children wore the accelerometers over their non-dominant hip, clipped around their waist during regular school hours (i.e., 8:00 A.M.–3:00 P.M.). Accelerometers were initialized to measure activity in 15-s epochs. Participant data were included in analyses if they recorded greater than 300 min (≥5 h) of monitoring time each day, for at least three days. Physical activity intensity categories (i.e., moderate-to-vigorous [MVPA], and vigorous [VPA]) and sedentary behavior were determined using age-specific cut-points for preschoolers: sedentary behavior 0–799 counts, MVPA ≥ 1680 counts, and VPA > 3368 counts [32]. Children’s average time per school day spent in MVPA, VPA, and sedentary behavior was calculated and used in the final data analysis. The Actigraph accelerometers were previously validated and have been used to assess preschooler’s physical activity [33].

### 2.5. Assessment of Cognitive Function

Children’s cognitive function was assessed using the Cambridge Neuropsychological Test Automated Battery (CANTAB^®^ [Cognitive assessment software] Research Suite 6, 2014). Specifically, the Motor Screening Task (MOT) and Reaction Time Task (RTI) were administered on a 10 × 10-inch iPad, where participants touch the screen and respond to the stimulus with their forefinger. The MOT, an index of psychomotor speed, measures (1) the time spent in correctly responding to the stimuli on screen during assessed trials (i.e., movement latency); (2) the accuracy of participants’ responses to touch the stimuli before they disappear (i.e., response errors). The RTI examines participants’ reaction time in both a simple (1 choice) and multiple choice (5 choices) format. Participants were instructed to hold down a home button with their dominant forefinger until a flashing button appeared (stimulus) and then, using the same finger, tap the flashing button and finally return to the home button. After the practice trials, the CANTAB^®^ software recorded participants’ reaction time (time between the stimulus and release of the home button) in both the simple and multiple-choice situations. Participants’ median score of simple reaction time and choice reaction time were recorded and used in the data analysis. Participants completed the MOT and RTI in one sitting (usually 8–10 min). CANTAB^®^ has been deemed reliable to generate consistent longitudinal test/retest outcomes while minimizing practice effects [34].

### 2.6. Statistical Analyses

The data analysis was conducted using the JASP Team statistical software (version 0.15; 2021). The descriptive statistics are reported as mean and standard deviation [SD] for MVPA, VPA, sedentary behavior (min/day), and for cognitive function variables (milliseconds) across the three time measures. A series of repeated measures of analysis of variance (ANOVA, IBM SPSS 28.0) were carried out to assess the mean differences in study variables over time (pre-test, post-test, and 1-month follow-up). Bonferroni-corrected pairwise comparisons were conducted for post hoc analyses of the time effect. Cohen’s d was used to detect the effect sizes (small = 0.2, moderate = 0.5, and large = 0.8) [35]. Two-tailed significant test was set up at 0.05 level.

## 3. Results

### 3.1. Overall Child Adherence and PedometerResults

Out of the original 13 children recruited, 11 participated (one child moved and one child dropped out) in all three time assessments and eight sessions of V-MAP and 10 children completed the cognitive function tests at three timepoints. During the eight sessions of the V-MAP intervention, preschoolers on average obtained 1105 steps in a 30-min recess session (36.85 steps/min).

### 3.2. V-MAP Effects on Physical Activity

**Figure 1:**
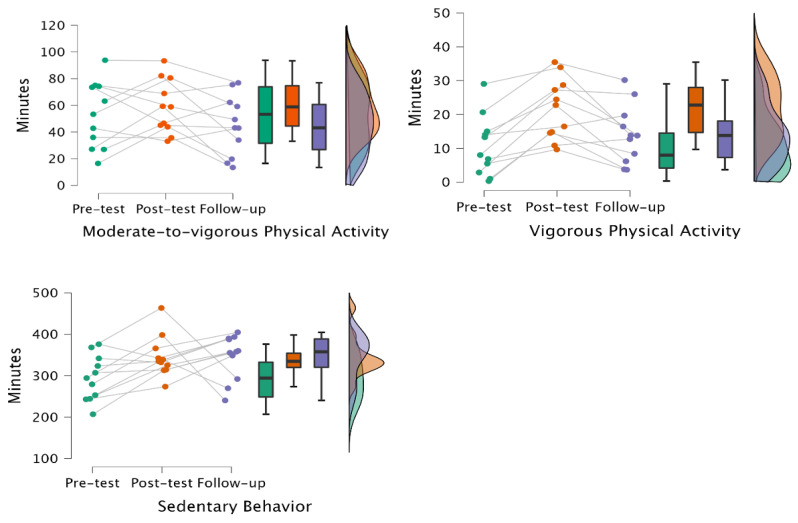
V-MAP Effects on Physical Activity and Sedentary Behavior.

Children’s physical activity and sedentary behavior across the three timepoints are presented in Table 2 and visualized in Figure 1. There was a significant increase in VPA (F (2, 20) = 14.04, *p* < 0.001; η^2^ = 0.58) after the V-MAP intervention. Although reduction in VPA was noticed at the 1-month follow-up compared to the post-test, the VPA measured at 1-month follow-up remained higher than the pre-test (14.06 min vs. 10.59 min; Cohen’s d = 0.49). Specifically, before the V-MAP intervention, preschoolers only engaged in 10.59 min in school-based VPA; while after the intervention, their VPA participation reached 21.75 min (t = 5.18, *p* < 0.001; Cohen’s d = 1.56). Children’s MVPA increased by 6 min after the intervention (52.97 min vs. 58.89 min; Cohen’s d = 0.32, small effect). Vigorous physical activity showed the largest change, with post-test values almost doubling baseline levels (Cohen’s d = 1.56, large effect). On average, the increments of MVPA were not sustained in the follow-up test, but VPA remained about 4 min greater than the pre-test. Sedentary behavior (SB) increased by ~51 min/day from pre- to post-intervention (294.36 to 345.66 min/day; Cohen’s d = 0.98, large effect) and remained almost the same at 1-month follow-up (345.38 min/day; Cohen’s d = 0.98 vs. pre, Cohen’s d ≈ 0.01 vs. post). In school-time terms, this corresponds to ~4.9 h/day (pre), ~5.8 h/day (post), and ~5.8 h/day (follow-up) during the monitored 8:00–15:00 window.

### 3.3. V-MAP Effects on Cognitive Function

**Figure 2:**
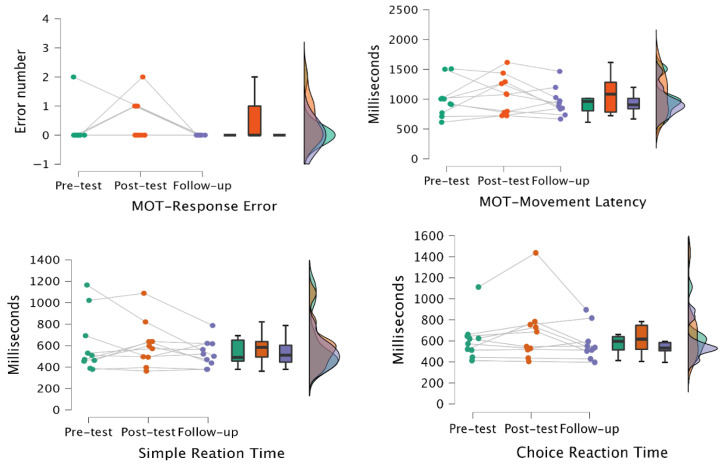
V-MAP Effects on Cognitive Function. (MOT = Motor Screening Task).

The impacts of V-MAP on preschoolers’ cognitive function at the three time measures are presented in Table 2 and visualized in Figure 2. Although we did not observe significant improvement in studied cognitive function variables (*p*s > 0.05) after the V-MAP intervention, some delayed effects were observed in the follow-up test. Specifically, at the 1-month follow-up test, all preschoolers showed improved psychomotor accuracy in MOT (i.e., reduced response errors: 0.02 vs. 0; Cohen’s d = −0.28) and their response speed (i.e., movement latency; 996.44 ms vs. 957.10 ms; Cohen’s d = −0.14) became faster compared with pre-and post-tests. These preschoolers also demonstrated an improvement in reaction time at the 1-month follow-up test for both simple reaction time tests (607.1 ms vs. 527.5 ms; Cohen’s d = −0.45) and the choice reaction time tasks (612.55 ms vs. 576.20 ms, Cohen’s d = −0.25), although there was no significant improvement immediately after completion of the V-MAP intervention.

## 4. Discussion

The purpose of the current study was to test the impact of a 6-week V-MAP intervention in recess on physical activity (i.e., sedentary behavior, MVPA, VPA) and cognitive function (i.e., movement latency, response errors, and reaction time) in Head Start preschoolers using a repeated measure with 1-month follow-up design. This exploratory trial provides preliminary evidence that implementing V-MAP in recess may contribute to preschoolers’ ability to achieve the recommended daily 60 min of MVPA. The results should be interpreted cautiously, as observed effects were modest and effects on MVPA were diminished by the one-month follow-up, although there were sustained benefits to VPA and delayed cognitive improvements at that timepoint. The findings provide meaningful insights that VR-integrated physical activity for as little as 5–10 min per day may yield benefits in cognitive function such as improved reaction time and reduced response errors.

The V-MAP intervention yielded, on average, 36.85 steps/min, which more than doubles previous reports of 12–16 steps/min of daily steps in preschoolers [36,37]. Presently, the U.S. physical activity guideline for preschoolers advises to achieve at least 60 min of structured MVPA per day. Previous studies [38,39] identified a practical step value of 9000 steps/day to equate to the 60-min MVPA guideline. Using this approximate total step value, 30 min of V-MAP contributed to over 12% of children’s total daily physical activity. By using this VR-integrated intervention approach, reaching this guideline may be more feasible and attainable for preschoolers. The increased steps from this VR-integrated intervention are consistent with a recent study [27]. That is, the preschoolers in the exergaming (i.e., non-immersive VR) group showed significantly more steps after intervention than children in the free-play, or non-gaming group [27,29]. Allocating funding toward VR-based gaming in federally funded preschool programs, like Head Start, may improve underprivileged children’s health trajectory, and position them towards school readiness when entering kindergarten.

The unique and novel finding of this study is the significant prolonged duration of vigorous-intensity physical activity seen in the post and follow-up tests after the 6-week V-MAP intervention. Obtaining vigorous physical activity in childhood may provide greater health benefits than just engaging in light and moderate physical activity, such as increased muscle mass, bone health, and aerobic capacity [40]. In grade-school level children (ages 8–17), studies have supported the notion that higher intensities of physical activity (i.e., vigorous, or strenuous) were more significant than lower intensities in predicting healthy weight status or adiposity [41,42,43]. A previous study [44] indicated that academic success (i.e., higher grades) was associated with students that participated in more vigorous physical activity, particularly the students who engaged for 20 min per day for at least 3 days per week. It is also known that physical activity participation declines over time and may start as early as 3–4 years old [45]. The current study provides initial evidence that from a stimulating 6-week VR-integrated physical activity intervention in recess, preschoolers acquired more vigorous activity than they were completing at baseline, and that was sustained even 1 month after the intervention was completed. Although V-MAP shows promise [46,47], access to non-immersive VR technology is far from guaranteed in most preschool contexts. Thus, this approach should be considered as a potential complementary strategy, rather than a replacement for more accessible physical activity promotion efforts (e.g., structured play, outdoor activities). In addition, introducing VR to young children requires caution because there is also a risk of fostering greater interest in screen-based gaming or creating frustration in settings where such technology is unavailable. Future work should carefully balance the benefits of VR with potential risks and ensure equitable access.

V-MAP also generated a 6-min increment of moderate-to-vigorous physical activity from pre-test to post-test. Similarly to vigorous intensity, MVPA is important during early childhood for developing fundamental movement skills, processing social-emotional and cognitive development [48,49]. Although the increase in MVPA was not sustained through the follow-up test, the acute MVPA effects of V-MAP are noteworthy due to the benefits these preschoolers may have attained, even for a short time. Implementing fundamental movement skill–based physical activity programs for as little as 5–10 min per day may help Head Start preschoolers achieve recommended MVPA. Because many of these children come from low-income families with limited opportunities for activity outside of school [50], school hours are a critical window [50]. Sedentary behavior showed little change across the V-MAP intervention, indicating that increases in physical activity did not necessarily reduce sitting time. Preschoolers inherently spend much of their school day in sedentary activities [51], and a 30-min recess bout of V-MAP may add VPA without displacing sedentary periods linked to teacher-led instruction or transitions. According to early-years guidelines [3,4], the focus should be on breaking up prolonged sitting and limiting recreational screen time (≤1 h/day for ages 3–4), while encouraging interactive, developmentally appropriate sedentary activities such as reading and storytelling. Preschool policies should therefore consider strategies to reduce overall sitting and integrate active breaks alongside programs like V-MAP. In our data, SB rose from ~4.9 h/day at baseline to ~5.8 h/day post-intervention and remained at ~5.8 h/day at follow-up during school hours. This pattern likely reflects structural features of the school context (e.g., scheduled seated lessons, limited active classroom breaks, indoor constraints) rather than an adverse effect of V-MAP per se. Increases in VPA can be offset by decreases in light-intensity activity rather than SB, leaving overall sitting time unchanged or even higher, if classroom routines remain primarily seated.

Even with this short intervention, improvements were observed in the cognitive function variables one month after completion of the intervention. At baseline testing, preschoolers exhibited more response errors than in the follow-up test; likewise, they also exhibited slower movement latency (response speed) in the baseline when compared to the follow-up test. This implies that preschoolers responded slower during incorrect responses, and quicker during correct responses, which is consistent with current literature [52]. During the V-MAP intervention, the games that were displayed on the pods were games that required accurate selection. For example, in “Seek the Smiley”, pods were arranged in a random semi-circle or in a staggered line. The smiley would only appear on one pod, while the rest had other shapes/objects. Children needed to make a quick, accurate selection before moving onto the next round. It is possible the improvement seen in errors and latency on the cognitive testing may be attributed to the consistent practice of selection (i.e., practice effects) throughout the 6-week intervention using SMARTfit^TM^. Even though values are not significant, the trend is favorable and more exploration using VR-integrated learning experiences is warranted, especially in an underserved pediatric population to advance their school readiness.

The delayed cognitive improvements observed at the one-month follow-up warrant deeper consideration. These results suggest that consolidation processes may occur over time, consistent with evidence that repeated motor-cognitive challenges can strengthen neural connections and support sustained attention and executive function [53,54]. Although non-significant immediately post-intervention, the observed improvements at follow-up may reflect a “lag effect” in which cognitive benefits emerge gradually. This finding has important implications for early education, suggesting that short, playful motor-cognitive interventions may contribute to longer-term readiness for academic learning. Lee and colleagues [55], however, found no significant relation of cognitive function and their 8-week fundamental movement skill-based after school program in their sample of early elementary children. Some authors have found success in significantly improving reaction time when using VR-based programming in special populations such as children with cerebral palsy or Autism [56,57]. For instance, Amprasi et al. [58] used a fully immersive VR-based program for 6 weeks in a cohort of 8–10-year-old typically developing children and found significant improvements in reaction time. Therefore, the findings of this study provide valuable insights that physical activity program targeting fundamental movement skills and technology integration (i.e., virtual reality) may be an effective and motivational approach for advancing health and development among early childhood populations. Future intervention studies should consider investigating these tactics together to determine optimal duration, frequency, and type.

While this study employed a longitudinal design with repeated measures to explore the effects of the V-MAP intervention, the small sample size and lack of a control group substantially limit the generalizability of the findings. These results should therefore be interpreted with caution and viewed as preliminary. Small-sample feasibility studies are common in VR-based interventions with young children, as prior research has also reported similar challenges in recruitment and retention [24,59]. Furthermore, emerging evidence suggests that VR exergaming can promote preschoolers’ physical activity and support cognitive functioning, particularly in areas such as attention, inhibitory control, and processing speed [60,61]. These studies, alongside our findings, indicate the potential of VR-based movement interventions as engaging tools to foster physical and cognitive development in early childhood. Lastly, using the iPad game-based neuropsychological assessment (CANTAB^®^) for evaluating cognitive function among young children is a strength of this study [36], but the results were highly variable for each child. Future work should employ larger, more diverse samples and include randomized controlled designs to strengthen the evidence base and improve generalizability.

## 5. Conclusions

Overall, the findings highlight the central role of recess as a critical window of opportunity for promoting physical activity and motor-cognitive engagement in preschoolers. This study provides preliminary evidence that allocating structured, engaging recess time may be one of the most feasible strategies to address disparities in early childhood health and development. The observed improvements in cognitive function should be further investigated to determine if the sustained improvements are consistent, and whether there are differential impacts of the intervention on activity duration and intensity. Overall, V-MAP may be an effective intervention that has potential to be implemented in other Head Start preschools to promote health and school readiness.

## Figures and Tables

**Table 1:** The V-MAP Intervention Game Descriptions.

SMARTfit^TM^ Games	SMARTfit^TM^ Game Descriptions	FMS Integration with SMARTfit^TM^ Play Pods
**Track the Targets—Dice color and shapes**	Track the Dice: strike “Go” to start. Hit each dice as it appears on pods.	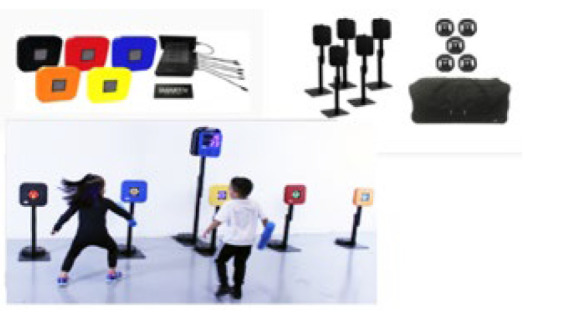 FMS Integration ❖ Locomotor skills: jumping, skipping, hopping, and running ❖ Object Control Skills: throwing, ball passing, and catching, striking Lesson Plan Sequence ❖ 2–5 min of warm-up ❖ 15–20 min of VR gaming with FMS integration ❖ 3–5 min of cool-down *Note:* Implement 2–3 skills/session within 2–3 SMARTfit^TM^ game
	2. Track the Shapes: strike “Go” to start. Hit each shape as it appears on pods.	
	3. Track the Colors: strike “Go” to start. Hit each color as it appears on pods.	
**Knock the Lights Out**	4. Lights Out Race: hit each light to turn off all pods.	
	5. Lights Out Keep 3 On: hit each light to turn off as many pods as you can before time runs out.	
**Track the Letters and Numbers**	6. Track the Numbers: hit the randomly selected number (1–9) then hit the next number in correct sequence.	
	7. Track the Letter: hit the letter “A” and then the next letter in correct sequence.	
**Seek the** **Smiley Face, Color, Letter**	8. Seek the Smiley Face: hit the smiley face until time runs out. 9. Seek the Color: hit the colored target until time runs out. 10. Seek the Letter: hit the letter “A” and continue through “Z” in sequence until time runs out.	

*Note:* V-MAP = Virtual Reality-infused Movement and Activity Program; FMS = Fundamental Movement Skills.

**Table 2:** Results of V-MAP Effects on Physical Activity and Cognitive Function.

Variables	Pre-Test (T1)	Post-Test (T2)	Follow-Up (T3)	T2 vs. T1	T2 vs. T3	T3 vs. T1
	*M* *(SD)*	*M* *(SD)*	*M* *(SD)*	*d*	*d*	*d*
MVPA	52.97 (24.90)	58.89 (20.17)	44.83 (22.43)	0.32	0.76	−0.44
VPA	10.59 (8.85)	21.72 (9.04)	14.06 (8.66)	1.56	1.08	0.49
SB	294.36 (54.85)	345.66 (50.04)	345.38 (54.47)	0.71	0.004	0.71
Simple RT	607.1 (272.49)	609.75 (213.55)	527.5 (125.45)	0.01	0.45	−0.45
Choice RT	612.55 (194.65)	682.25 (297.04)	576.2 (159.32)	0.48	0.73	−0.25
Response Error	0.2 (0.63)	0.5 (0.71)	0 (0)	0.41	0.69	−0.28
Movement Latency	996.44 (300.47)	1080.27 (319.49)	957.1 (232.76)	0.29	0.43	−0.14

*Note:* MVPA = moderate-to-vigorous physical activity; VPA = vigorous physical activity; SB = Sedentary Behavior. T1 = Time-1 measures, T2 = Time-2 measures, T3 = Follow-up measures.

## Data Availability

The data are not publicly available due to ethical and privacy restrictions to protect participant confidentiality, but are available from the corresponding author upon reasonable request.
